# CircRNA_101237 promotes NSCLC progression via the miRNA-490-3p/MAPK1 axis

**DOI:** 10.1038/s41598-020-65920-2

**Published:** 2020-06-03

**Authors:** Zhi-ye Zhang, Xiao-hui Gao, Ming-ying Ma, Chun-ling Zhao, Ya-li Zhang, Shuang-shuang Guo

**Affiliations:** Department of Oncology, First Affiliated Hospital, College of Clinical Medicine, Henan University of Science and Technology, Henan province, 471000 China

**Keywords:** Cancer genetics, Cell invasion

## Abstract

Non-small cell lung cancer (NSCLC) is a common type of lung cancer, characterized by a poor prognosis. In the last several years, more and more studies have demonstrated the significant roles played by circular RNAs (circRNAs) in different human tumors progression including NSCLC. The present study was to explore the mechanism of hsa_circ_101237 in regulating non-small cell lung cancer (NSCLC). Totally 303 NSCLC cases were enrolled. A549 and H1299 cells were transfected. Cells viability, migration and invasion were determined by CCK-8 assay and transwell experiment, respectively. Luciferase reporter gene assay and RNA immunoprecipitation (RIP) assay were performed. hsa_circ_101237, miR-490-3p and MAPK1 expression in tissues/cells were detected by qRT-PCR. The study found an elevation in the expression of Hsa_circRNA_101237 in both NSCLC tissues and cell line. High Hsa_circRNA_101237 expression predicted poor survival in NSCLC. Meanwhile, we found that hsa_circRNA_101237 expression sponged miR-490-3p to enhance MAPK1 expression, thus significantly promoting NSCLC cell lines proliferation, migration, and invasion. MAPK1 restoration prevented NSCLC cells proliferation, migration, and invasion to be repressed due to hsa_circRNA_101237 knockdown. To sum up, as revealed by the study, hsa_circRNA_101237 promoted the expression of MAPK1 via miRNA-490-3p sponge, thus affecting the NSCLC as an important onco-circRNA.

## Introduction

Lung cancer, characterized by a poor prognosis but a high morbidity, is a common malignant tumor, non-small cell lung cancer (NSCLC) are identified as the most common type of lung cancer (occupying about 85%)^1^. As is well known, smoking is the main cause of NSCLC^2^. As NSCLC does not show obvious clinical symptoms and the screening programs are not effective, most patients with NSCLC, once they are diagnosed, are at advanced stages with a poor prognosis^3^. More and more clinical studies found that metastasis greatly hinders the treatment of NSCLC cancer; therefore, in-depth understanding the metastasis mechanisms is beneficial for effectively treating NSCLC.

Circular RNAs (circRNAs) are a kind of non-coding RNAs with endogenous and conserve, forming a covalently closed continuous loop via back-splice without 3ʹ-end or 5ʹ-end^4^. Due to the feature, circRNAs exhibit a lot of properties, and many properties were found not long ago. circRNAs have a special closed loop structure, making them able to resist the degradation medicated by exonuclease. On that account, they are more stable compared with most other linear RNAs, thus they can be used as a biomarker to effectively diagnose and treat cancers^5^. circRNAs can interact with RNA-binding proteins, so as to regulate target gene expression^6^. Besides, it has been indicated that circRNAs can be sinks for miRNAs, to control the function processed by miRNAs^7^. Many human cancers see altered circRNA expression, and many researches have revealed the key role played by circRNAs in the tumorigenesis. circRNA_101237, a new circRNA identified recently, whose encoding gene is located at chromosome (chr) 13:26974589-26975761 and which is produced by backsplicing of exons 10, 11 and 12 of cyclin-dependent kinase (CDK) 8, has been reported to be implicated in Cisplatin resistance-associated of HCC^8^. Nevertheless, the function and mechanism of hsa_circ_101237 in regulating NSCLS remain unknown.

The study focused on illuminating how hsa_circRNA_101237 affects the pathogenesis of NSCLC, as well as the regulating mechanism *in vitro*. In terms of the function, hsa_circRNA_101237 facilitated the development, the migration and the invasion of NSCLC cell. In terms of the mechanism, hsa_circRNA_101237 regulated the miR-490-3p/MAPK1 axis, and contributed to NSCLC progression.

## Materials and methods

### Patients and tumor tissues

We obtained 303 snap-frozen NSCLC tissues together with paired nearby non-tumorous tissues in total from NSCLC patients. The study has gained the informed consent of these patients prior to study, and been approved by the ethics committee of the First Affiliated Hospital of Henan University of Science and Technology. The performance of the study followed the guidelines of the committee and the Declaration of Helsinki. Table 1 lists patients’ demographics and clinical findings. All experiments were performed in accordance with the relevant guidelines and regulations.

**Table 1:** Association between circRNA_101237 expression and clinicopathological features of human NSCLC.

Clinical features	Total	circRNA_101237		*p*-vaule
		High (N = 153)	Low (N = 150)	
**Age (years)**				0.252
<60	90	50	40	
≥60	213	103	110	
**Gender**				0.154
Male	170	92	78	
Female	133	61	72	
**Smoke**				0.232
No	68	30	38	
Yes	235	123	112	
**Drink**				0.118
No	143	79	64	
Yes	160	74	86	
**Tumor size (cm)**				0.044
<5	122	53	69	
≥5	181	100	81	
**Differentiation grade**				0.144
Well	101	45	56	
Moderate + Poor	202	108	94	
**TNM stage**				0.001
I + II	191	83	108	
III	112	70	42	
**Lymph node metastasis**				0.003
No	191	84	107	
Yes	112	69	43	
**CEA, ng/ml**				0.230
<5	145	68	77	
≥5	158	85	73	
**CA19-9, kU/L**				0.310
<40	128	69	59	
≥40	175	84	91	

CA19-9 carbohydrate antigen 19-9; CEA, carcinoembryonic antigen; Pearson chi-square test was used for comparison between subgroups.

### Quantitative real-time polymerase chain reaction (qRT-PCR)

Total RNA was isolated from cells and tissues by virtue of the Trizol reagent (Invitrogen). TaqMan MicroRNA reverse transcription kit (Applied Biosystems, Foster City, CA) was employed to perform cDNA synthesis for miR-490-3p. One Step PrimeScript cDNA kit (Qiagen, Hilden, Germany) was employed to perform cDNA synthesis for MAPK1 and hsa_circ_101237. The GeneAmp 7500 system (Applied Biosystems) was adopted to perform qRT-PCR in triplicate for determining the expression of MAPK1 and hsa_circ_101237. miR-490-3p expression was also evaluated via the TaqMan MicroRNA assay. GAPDH and U6 were regarded as the endogenous reference gene for the MAPK1 and hsa_circ_101237 and the loading control for the miR-490-3p, respectively. The 2^−ΔΔCT^ method helped to assess the relative expression exhibited by MAPK1, miR-490-3p and hsa_circ_101237.

### RNase R treatment

The total RNAs were treated via the RNase R (Epicentre Technologies). In brief, experimenters divided the RNAs aliquots extracted from A549 cells and H1299 cells into 2 parts: one was for the digestion of RNase R and another was only for the control group with digestion buffer. As for the first part, we mixed the total RNA (2 μg) with 2 μl 10 × RNase R Reaction Buffer and 2 μl RNase R (20 U/μl); as for the second part, DEPC-treated water was used to replace the RNase R. Subsequently, RNA samples received 30 minutes of incubation at 37 °C water. qRT-PCR helped to analyze the detected GAPDH mRNA and hsa_circ_101237. RNA treated with RNase R helped to detect the resistance exhibited by hsa_circ_101237 to the RNase R exonuclease digestion.

### Cell culture and transfection

The human embryonic lung fibroblasts (MRC-5) cells and the NSCLC cell lines (A549 cells and H1299 cells) were provided by the American Type Culture Collection (ATCC, Rockville, MD). Cells received incubation treatment in RPMI-1640 (Solarbio, Beijing, China) and dulbecco’s modified eagle medium (DMEM; Solarbio) composed of 1% penicillin/streptomycin (Solarbio) and 10% fetal bovine serum (FBS; Solarbio) under 5% CO2 at 37 °C. Plasmids and viruses involved in this paper were all purchased from GENEWIZ (Jiangsu, China). Lipofectamine 2000 (Invitrogen, Carlsbad, CA, USA) was employed to transfect abovementioned plasmids into A549 cells and H1299 cells. 48 hours after the transfection, we harvested cells for later study.

### Colony formation assay

Each group of treated cells (1 × 10^3^ per well) was seeded into 10 cm culture dish, and cultured for 2 weeks. Cells underwent culture treatment under 5% CO2 at 37 °C in RPMI-1640 composed of 80 U/ml penicillin and 20% FBS and 100 µg/ml streptomycin (Gibco; Thermo Fisher Scientific, Inc.). Finally, 1% crystal violet was used to stain these colonies, followed by number counting.

### Transwell migration & invasion assay

During the migration assay, we seeded H1299 and A549 cells that were suspended in medium free of serum on the top chamber after the transfection, and added medium that had 10% FBS into the lower chamber. During the invasion assay, Matrigel (BD, Franklin Lakes, NJ, USA) were coated on the transwell inserts (Fisher Scientific, Waltham, MA, USA). After cells were incubated for 24 hours, a cotton swab was used to remove cells on transwell member’s upper surface gently and methanol helped to fix cells on transwell member’s lower surface, then 0.5% crystal violet (Solarbio) was used to stain these cells, followed by being counted from 5 microscopic fields under random selection.

### Cell proliferation capacity detection

We plated H1299 cells and A549 cells in 96-well plates at 2 × 103 cells/well and let them grow in 10% FBS medium for 24 hours. Following the transfection, each well was added with cell count kit-8 (CCK-8) solution (10 μl, Beyotime, Shanghai, China) and cells underwent 2 hours of incubation at 37 °C in a incubator with 5% CO2. A spectrophotometer (Bio-Rad, Hercules, CA, USA) helped to read each well’s absorbance at 450 nm.

### Luciferase reporter assay

We first amplified the wild-type (WT) sequence of the has-circ_101237 together with the WT 3ʹUTR sequence of the MAPK1 that contained predicted binding site of the miR-490-3p and then subcloned them into the pGL3 Basic reporter vectors (Promega, Madison, WI, USA) by virtue of Nhe I and Xho I restriction sites, aiming at generating pGL3-circ_101237-WT vector and pGL3-MAPK1 3ʹUTR vector. Quick Change Lightning kit (Stratagene, La Jolla, CA, USA) was adopted to conduct site-directed mutagenesis, for constructing mutant-type (MUT) sequence of the has-circ_101237. A549 cells and H1299 cells were under transfection of the pGL3-circ_101237-MUT, pGL3-circ_101237-WT, or pGL3-MAPK1 3ʹUTR, together with the miR-490-3p or the matched controls. Cell collection was performed 48 hours after the transfection and Dual-Luciferase Assay System (Promega) was adopted to analyze these collected cells, following the instruction of manufacturer.

### CircRNA localization

To localize circRNA, Cytoplasmic & Nuclear RNA Purification Kit (Amyjet) helped to isolate and extract the nuclear and cytoplasmic RNA of A549 cells, and qRT-PCR helped to measure the expression of circ_101237 in above two types of RNA. GAPDH was the cytoplasm control and U1 was the nuclear control.

### Statistical analysis

SPSS 17.0 (SPSS Inc., IL, and USA) helped to analyze above results, which were expressed as means ± SD. Student’s t-tests together with proper one-way ANOVAs were conducted to compare data. The chi-squared test assisted in analyzing how hsa_circ_101237 expression affects the clinical findings of patients. Kaplan-Meier curves together with log-rank tests were employed to compare the survival outcomes. Factors related to patient’s outcomes were identified via the univariate regression analyses, with significant ones being included into the multivariate analysis. All these experiments were performed repeatedly in triplicate, and P < 0.05 was considered significant.

## Results

### Hsa_circ_101237 is increased in NSCLC

**Figure 1:**
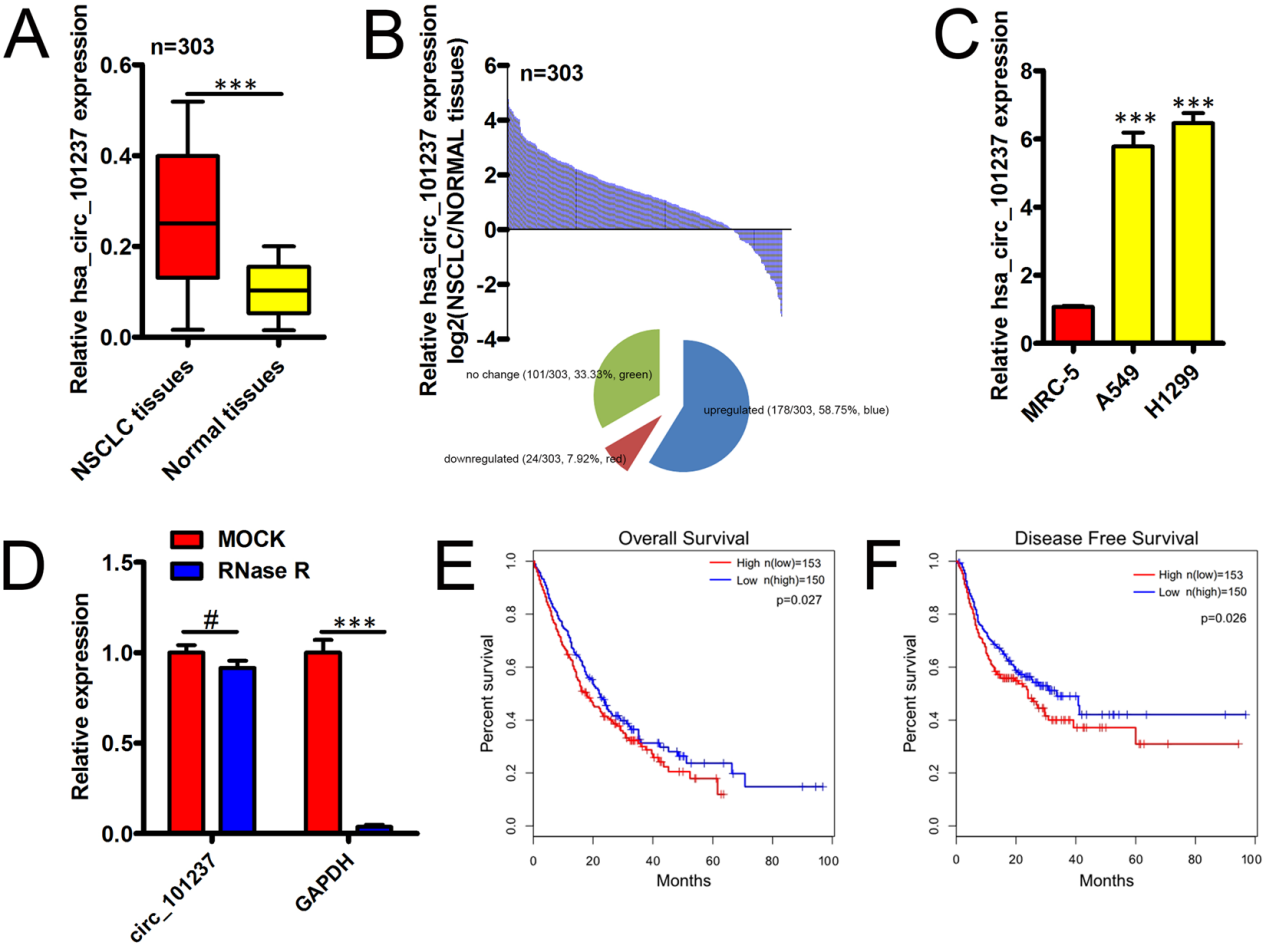
hsa_circ_101237 expression in NSCLC tissues and cell lines, and its clinical significance. (**A**) Determination of hsa_circ_101237 expression in 303 paired samples of the NSCLC tissues. (**B**) Proportion pie chart and histogram of NSCLC sample that saw the up-regulation of hsa_circ_101237 expression (242/303, 79.87%, red), down-regulation (33/303, 10.89%, blue), or no change (28/303, 9.24%, brown). Expression value of Log2 (T/N) > 1 and < −1 were regarded as as higher and lower expression, respectively, and that in the range of −1–1 was regarded as no obvious change. (**C**) Based on the qRT-PCR analysis on hsa_circ_101237 expression, hsa_circ_101237 presented up-regulation in NSCLC cells. (**D**) qRT-PCR helped to determine hsa_circ_101237 expression after RNase R treatment in A549 cell line. (**E**,**F**) The prognosis presented by NSCLC patients whose hsa_circ_101237 levels were different was assessed via Kaplan–Meier survival analysis. The cutoff value referred to median hsa_circ_101237 expression level in those NSCLC tissues. *P < 0.05; **P < 0.01; ***P < 0.001.

Firstly, qRT-PCR assay was conducted aiming at characterizing the hsa_circ_101237 in NSCLC tissues, which demonstrated that NSCLC tissues held an obviously higher hsa_circ_101237 expression compared with nearby non-tumor tissues (Fig. 1A,B). In addition, hsa_circ_101237 expression remarkably increased in NSCLC cells, represented by A549 cells and H1299 cells, in comparison with MRC-5 cells (Fig. 1C). circRNAs exhibited a resistance to the degradation mediated by exonuclease. RNase R was employed to treat the total RNA from the NSCLC cell lines, and results found the resistance of hsa_circ_101237 to RNase R, however, RNase R could degrade GAPDH mRNA (Fig. 1D).

### Hsa_circ_101237 is correlated with poor prognosis in NSCLC

Also, to assess the clinical significance of hsa_circ_101237, we evaluated the association between its expression and clinic-pathological parameters. Then patients were divided into two groups, group with high hsa_circ_101237 expression (n = 153) and group with low hsa_circ_101237 expression (n = 150) considering median circRNA expression level, for further examining the way that hsa_circ_101237 expression affects the NSCLC progression. Subsequently, these patients’ clinical findings were compared, which demonstrated that hsa_circ_101237 expression could greatly increase under the impact of and lymph node metastasis (p = 0.006), large size of tumor (p = 0.024), and advantaged TNM stage (p = 0.025) (Table 1). We next analyzed the prognostic relevance of hsa_circ_101237 levels in this NSCLC patient cohort. We found that the median OS in group with high hsa_circ_101237 expression was obviously shorter (P = 0.027, Fig. 1E). Similar findings were also observed with respect to patient disease-free survival (DFS, P = 0.026, Fig. 1F). This thus suggested that expression of high hsa_circ_101237 is positively correlated with patient prognosis such that elevated expression of hsa_circ_101237 is linked with poorer patient outcomes. In an additional multivariate analysis (Table 2), we confirmed a significant relationship between the expression of hsa_circ_101237 and NSCLC patient OS (HR = 1.311, 95% CI: 1.121-2.269, p = 0.042) and DFS (HR = 1.436, 95% CI: 1.266–2.107, p = 0.046).

**Table 2:** Univariate and multivariate Cox regression analyses of overall survival and disease-free survival in NSCLC patients.

Variables	Univariate analyses			Multivariate analyses		
	Hazard ratio	95% CI	P	Hazard ratio	95% CI	P
**Overall survival**						
Age (years) ≥ 60/<60	1.115	0.432–2.297	0.187	—	—	—
Gender Male/female	1.439	0.398–1.849	0.223	—	—	—
Smoke Yes/No	1.322	0.621–2.083	0.098	—	—	—
Drink Yes/No	1.059	0.321–1.709	0.628	—	—	—
Tumor size (cm) ≥ 5/<5	1.589	1.129–2.745	0.028	1.644	1.346–2.833	0.044
Differentiation grade Poor+ moderate / well	1.526	1.255–2.218	0.029	1.578	1.256–2.672	0.026
TNM stage III/I + II	2.137	1.437–2.987	0.003	1.416	1.178–2.564	0.039
Lymph node metastasis Yes/no	1.499	1.253–2.832	0.016	1.528	1.211–2.183	0.049
CEA, µg/ml ≥ 4.5/<4.5	1.319	0.832–2.471	0.294	—	—	—
CA19–9, kU/L ≥ 40/<40	1.269	0.893–2.941	0.462	—	—	—
CircRNA_101237 expression High/Low	2.379	1.403–3.116	0.027	1.311	1.121–2.269	0.042
**Disease-free survival**						
Age (years) ≥ 60/<60	1.156	0.042–1.834	0.405	—	—	—
Gender Male/female	1.392	0.348–1.725	0.686	—	—	—
Smoke Yes/No	1.344	0.554–2.153	0.232	—	—	—
Drink Yes/No	1.434	0.336–2.503	0.174	—	—	—
Tumor size (cm) ≥ 5/<5	1.475	1.356–2.366	0.008	1.596	1.231–2.794	0.026
Differentiation grade Poor+ moderate / well	1.632	1.451–2.573	0.015	1.432	1.287–2.432	0.031
TNM stage III/I + II	1.796	1.246–2.903	0.005	1.585	1.322–2.226	0.025
Lymph node metastasis Yes/no	1.832	1.566–3.011	0.034	1.657	1.465–2.114	0.019
CEA, µg/ml ≥ 4.5/<4.5	1.385	0.856–1.877	0.112	—	—	—
CA19–9, kU/L ≥ 40/<40	1.257	0.438–1.790	0.073	—	—	—
CircRNA_101237 expression High/Low	1.658	1.235–2.456	0.029	1.436	1.266–2.107	0.046

CA19-9 carbohydrate antigen 19-9; CEA, carcinoembryonic antigen; HR, hazard ratio; 95% CI, 95% confidence interval.

### Hsa_circ_101237 knockdown hinders the proliferation, the migration and the invasion of NSCLC cells

**Figure 2:**
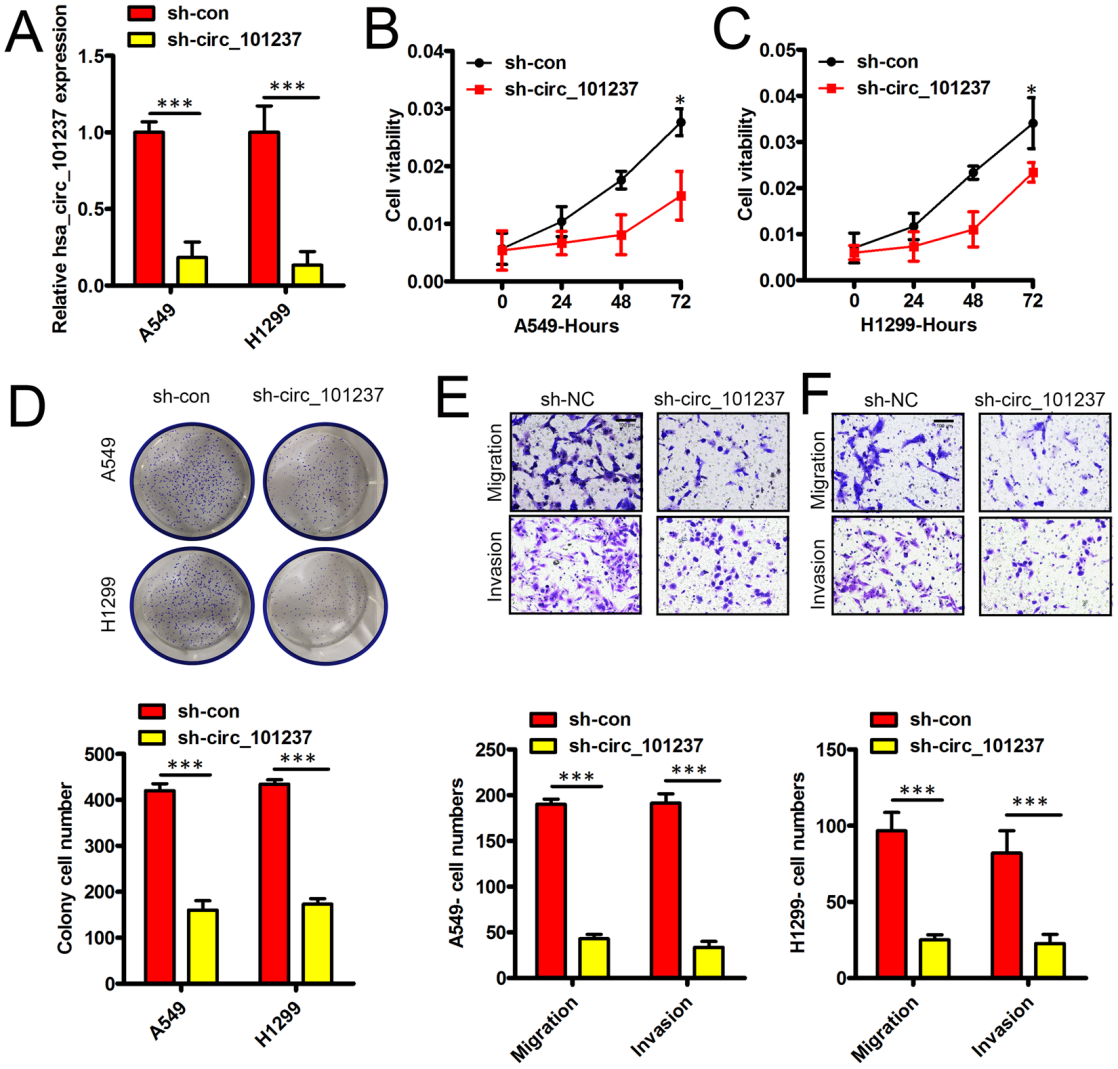
hsa_circ_101237 knockdown hinders the proliferation, the migration and the invasion of NSCLC cells. (**A**) Hsa_circ_101237 level was detected by qRT-PCR in sh-con or sh-circ_101237 transfected cells. (**B**,**C**) Evaluation of the viability exhibited by cell was performed via the CCK-8 assay in cells transfected by sh-con or sh-circ_101237. (**D**) Colony Formation Assay helped determine the impact of hsa_circ_101237 on colony formation ability. (**E**) Transwell migration assay helped determine the migration of H1299 cells and A549 cells following the transfection. (**F**) Transwell invasion assay aimed at analyzing the invasion process of H1299 cells and A549 cells under the transfection of sh-con or sh-circ_101237. *P < 0.05; **P < 0.01; ***P < 0.001.

Considering the up-regulation of hsa_circ_101237 in NSCLC, hsa_circ_101237 was knocked down for investigating the biological function owned by hsa_circ_101237 in NSCLC by using sh-circ_101237 to transfect A549 cells and H1299 cells. In comparison with the control, transfecting sh-circ_101237 could greatly lower hsa_circ_101237 expression in above two cells (Fig. 2A). The CCK-8 assay confirmed that hsa_circ_101237 down-regulation significantly weakened the viability exhibited by above two cells (Fig. 2A,C). Hsa_circ_101237 knockdown weakened the colony formation ability of above two cells (Fig. 2D). Besides, hsa_circ_101237 knockdown significantly hindered the migration process of above two cells (Fig. 2E). Transwell invasion assay also helped to confirm that hsa_circ_101237 knockdown could inhibit cell invasion (Fig. 2F). As found by above data, hsa_circ_101237 knockdown assisted in inhibiting the proliferation, the migration and the invasion of NSCLC cells.

### Hsa_circ_101237 sponges miR-490-3p to facilitate NSCLC development

**Figure 3:**
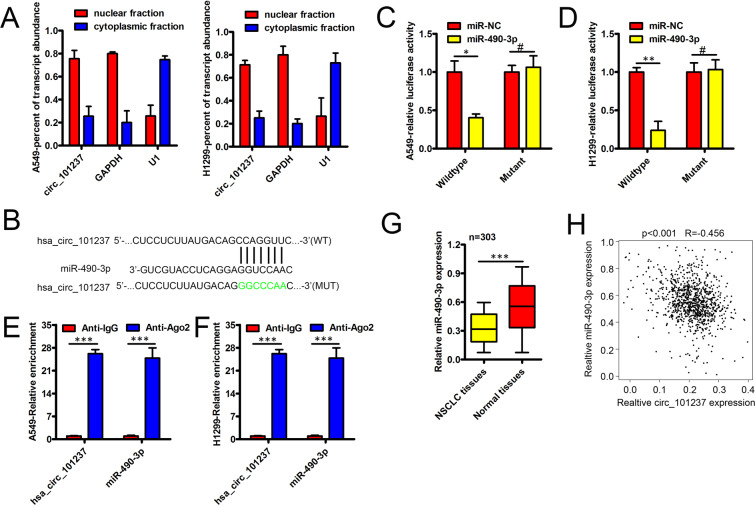
Hsa_circ_101237 sponges miR-490-3p to facilitate NSCLC development. (**A**) As shown in nuclear RNA fractionation and cytoplasmic experiments, hsa_circ_101237 could be mainly found in cytoplasm of H1299 cells and A549 cells. (**B**) The predicted binding site of miR-490-3p in hsa_circ_101237 3ʹUTR together with the mutated sites. (**C,D**) Co-transfection of miR-con or miR-490-3p and luciferase reporter vectors into H1299 cells and A549 cells. (**E,F**) RIP assay was conducted using the Ago2 and IgG antibody to immunoprecipitate. The expression of hsa_circ_0087862 and miR-490-3p was detected by qRT-PCR in H1299 cells and A549 cells. (**G**) qRT-PCR shew the expression of miR-490-3p in the NSCLC tissues and normal tissues nearby. (**H**) The expression correlation between miR-490-3p and Hsa_circ_101237 in 303 NSCLC tissues was determined by Pearson’s correlation analysis. *P < 0.05; **P < 0.01; ***P < 0.001.

As revealed by a lot of studies, via miRNA sponge, circRNAs play the role of a major regulator, which blocks silencing complex mediated by Ago2 as ceRNAs^9,10^. For figuring out if hsa_circ_101237 could be a ceRNA, its subcellular localization was firstly detected. Nuclear/cytoplasmic fraction assay results showed that, most hsa_circ_101237 presented a preferential localization in cytoplasm (Fig. 3A). Therefore, it was assumed that hsa_circ_101237 could be a ceRNA. Under the predication of bioinformatics analyses, it is possible for complementary base in miR-490-3p ranked the top to act as a direct target of hsa_circ_101237 (Fig. 3B). Besides, as verified by assays, wild-type hsa_circ_101237 greatly hindered the luciferase activity exhibited by miR-490-3p instead of the mutant form (Fig. 3C,D). Based on an immunoprecipitation assay conducted on NSCLC cells also helped to verify that hsa_circ_101237 could target miR-490-3p in a direct manner (Fig. 3E,F). Meanwhile, miR-490-3p expression remarkably decreased in A549 cells and H1299cells comparison with MRC-5 cells (Fig. S1A). Additionally, expression of miR-490-3p was lower in NSCLC tissues compared with non-tumor tissues (Fig. 3G, p < 0.001). The expression of miR-490-3p and hsa_circ_101237 in NSCLC tissues exhibited a negative correlation (Fig. 3H, R = − 0.456, p < 0.001). Above data demonstrated that as a competing endogenous RNA, hsa_circ_101237 could interact with miR-490-3p, thus inhibiting their action. Rescue experiments were used to further elucidate whether the activities of hsa_circ_101237 in NSCLC cells were mediated by the miR-490-3p. First, si-hsa_circ_101237 was co-transfected with miR-490-3p inhibitor into NSCLC cells, and cell proliferation, migration, and invasion were examined. The silencing of circ_101237 inhibited NSCLC cell proliferation (Fig. S2A), and impaired cell migration (Fig. S2B) and invasion (Fig. S2C). Meanwhile, miR-497-5p inhibition partially neutralized the effects of circ_101237 silencing in these cells (Fig. S2A–C).

### MiR-490-3p inhibits NSCLC progression via MAPK1

**Figure 4:**
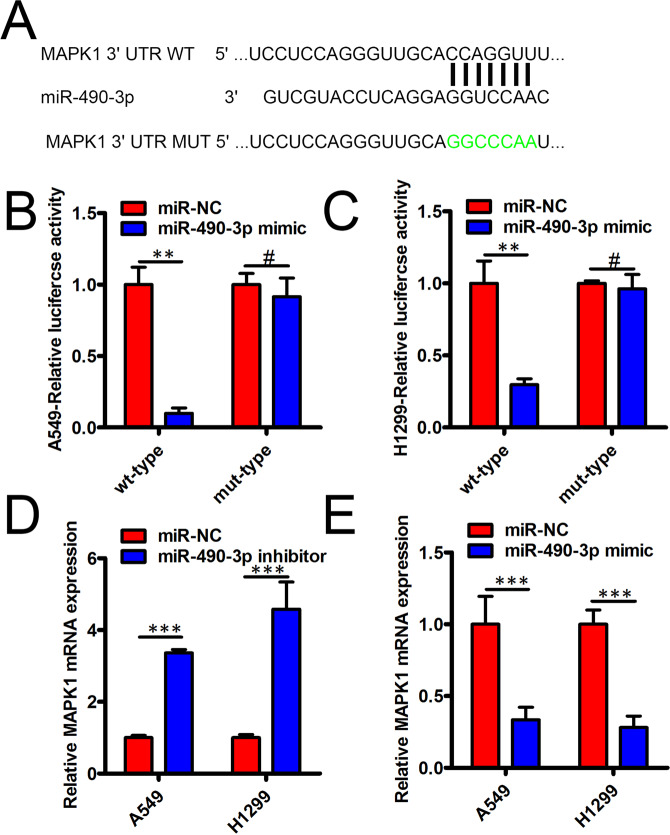
miR-490-3p inhibited NSCLC progression via MAPK1. (**A**) Bioinformatics tools helped to find that miR-490-3p and MAPK1 3ʹ-UTR saw the existence of complementary binding sites. (**B,C**) Luciferase reporter assay confirmed that miR-490-3p and MAPK1 3ʹ-UTR exhibited molecular binding in A549 cells (**B**) and H1299 cells (**C**). (**D,E**) qRT-PCR assay found the existence of mRNA MAPKP level in indicated NSCLC cells. *P < 0.05; **P < 0.01; ***P < 0.001.

According to ceRNAs theory^10^, it is necessary for hsa_circ_101237 and mRNA to share miR-490-3p, thereby releasing the repression of mRNA through a competitive binding with the miR-490-3p. TargetScan was used to perform bioinformatics analysis, MAPK1 was found to share complementary binding sites with miR-490-3p (Fig. 4A), and it was selected for further verification because this MAPK1 was reported to act as an oncogene during NSCLC progression^11^ and a previous study has indicated that MAPK1 is a direct target gene of miR-490-3p in esophageal squamous cell carcinoma cells^12^. A luciferase reporter assay helped to verify the complementary binding association between them (Fig. 4B,C). MAPK1 mRNA expression presented a significant increase via miR-490-3p inhibition in indicated NSCLC cell lines (Fig. 4D). Meanwhile, MAPK1 mRNA expression presented a significant decrease via miR-490-3p mimic in NSCLC (Fig. 4E). We next analyzed the prognostic relevance of MAPK1 levels in this NSCLC patient cohort. 303 NSCLC patients were divided into two groups, group with high MAPK1 expression (n = 152) and group with low MAPK1 expression (n = 151) considering median MAPK1 expression level. We found that MAPK1 expression was no obviously associated with NSCLC patients survival (DFS, P = 0.43; DFS, P = 0.062, Fig. S3A,B).

### MAPK1 over-expression hinders the way that hsa_circ_101237 knockdown affects the biological behavior exhibited by NSCLC cells

**Figure 5:**
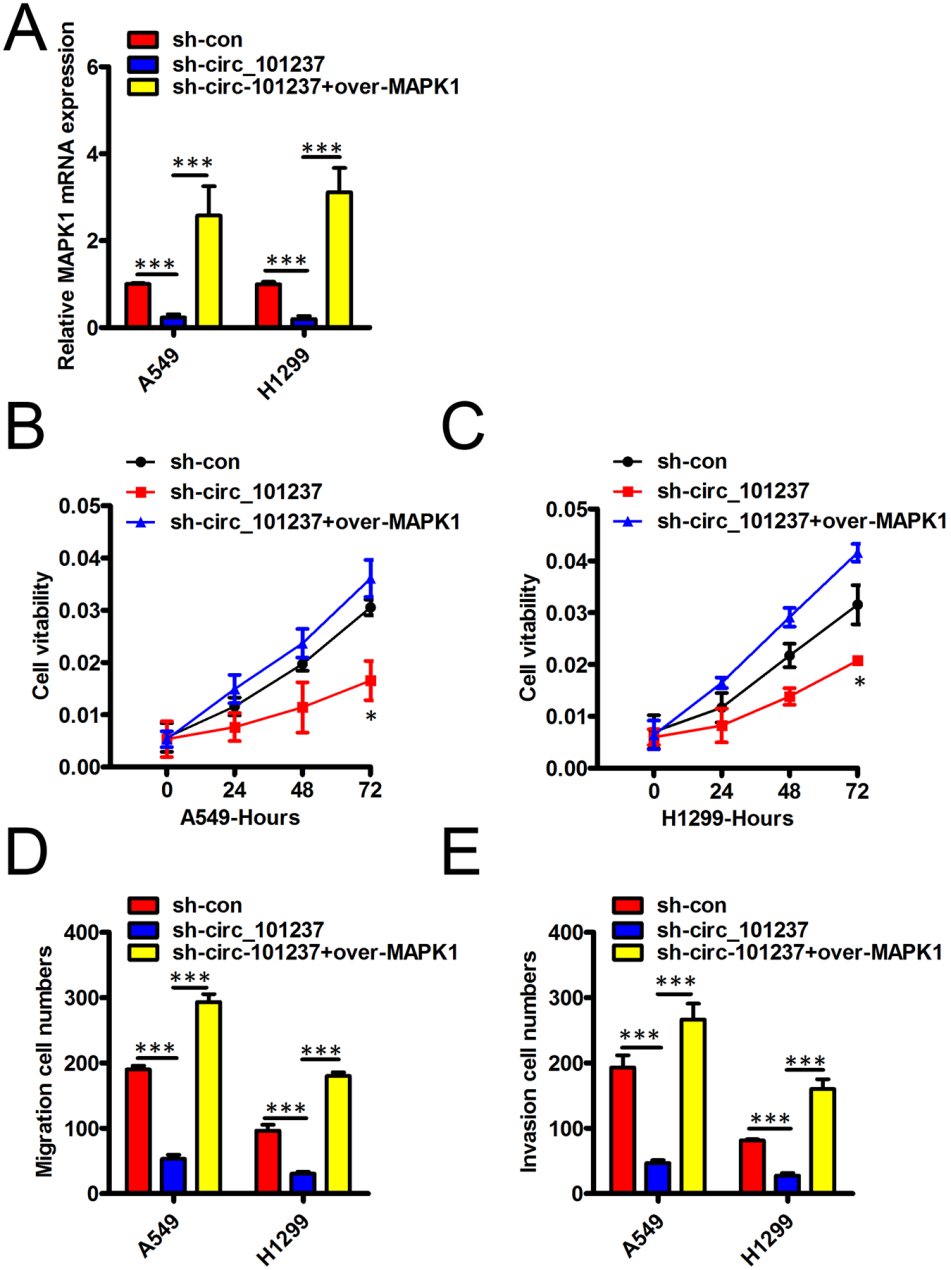
MAPK1 over-expression hinders the way that hsa_circ_101237 knockdown affects the biological behavior exhibited by NSCLC cells. (**A**) qRT-PCR assay together with Western blot assays helped determine the MAPK1 mRNA levels in A549 cells and H1299 cells under the transfection of sh-con, sh-circ_101237 alone or the pcDNA-MAPK1. (**B**,**C**) CCK-8 assay was conducted to measure the viability exhibited by A549 cells and H1299 cells at 24 h, 48 h, 72 h and 96 h following the transfection. (**D**,**E**) Transwell migration assay was performed to detect the migration and the invasion of cells in A549 cells and H1299 cells under the transfection of sh-con, sh-circ_101237 alone or the pcDNA-MAPK1. *P < 0.05; **P < 0.01; ***P < 0.001.

Next, we wonder whether hsa_circ_101237 could enhance NSCLC cell proliferation, migration and invasion ability via MAPK1. Firstly, Fig. 5A demonstrated the ability of has-circ_101237 knockdown to greatly lower the mRNA MAPK1 level in A549 cells and H1299 cells, and the ability could be greatly reversed once MAPK1 was over-expressed. As displayed by the CCK-8 assay, as has-circ_101237 was down-regulated, the cell viability exhibited by the two cells would be greatly weakened, and the situation could be effectively mitigated if MAPK1 was up-regulated (Fig. 5B,C). Also, has-circ_101237 silencing hindered the migration and the invasion process of the two cells, while once MAPK1 was over-expressed, above impacts could be hindered (Fig. 5D,E).

## Discussion

circRNAs first appeared in the 1970s^13^. In the original studies, viral genetic materials provided evidences about circRNAs^14–16^. Later, as reported, a large number of genes could generate individual circRNAs, including SRY, ETS-1, P450, etc. in various cells. Even so, biological functions of circRNAs are unclear^17–20^. In 2012, high-throughput RNA sequencing analysis together with bioinformatics analysis helped to disclose human circRNAs in various tissues and cells^21,22^. Since that time, research on circRNA has begun to focus on elucidating its impact on human diseases, with cancer included. As reported by a lot of studies, there is a correlation between abnormally expressed circRNAs and clinical features, with the former possibly becoming a biomarker for NSCLC^23–25^. circRNA_101237, a novel identified circRNA, whose encoding gene is located at chromosome (chr) 13:26974589-26975761 and which is produced by backsplicing of exons 10, 11 and 12 of cyclin-dependent kinase (CDK) 8, has been reported to be implicated in Cisplatin resistance-associated of HCC^8^. Nevertheless, we still do not well understand how circRNA_101237 affects NSCLC as well as the potential mechanisms it holds. The study found an obvious up-regulation of circRNA_101237, a kind of circular RNA in NSCLC tissues. High expression of circRNA_101237 positively correlated with lymph node metastasis, large size of tumor, and advantaged TNM stage as well as predicted poor prognosis specific to patients suffering NSCLC.

As proposed by the ceRNA hypothesis, miRNA response elements for RNA transcripts are the same, which compete for binding to the miRNAs, thus the expression is modulated mutually^26^. Here, miRNAs that bind with circRNA_101237 were screened by bioinformatic analyses together with the pull-down assays of circRNA_101237. At the same time, circRNA_101237 luciferase reporter was designed for identifying how circRNA_101237 directly interacted with miRNA. Accordingly, in the pull-down assay of circRNA_101237, miR-490-3p exhibited the largest enrichment and miR-490-3p was capable of reducing the luciferase activity possessed by circRNA_101237 luciferase reporter. Based on this, circRNA_101237 could be a ceRNA to bind to miR-490-3p in terms of the mechanism.

Specific to circRNA mechanism acting as ceRNA, the circRNA-miRNA-mRNA regulatory network about hsa_circ_101237 was explored, finding the ability of hsa_circ_101237 to facilitate MAPK1 expression through miR-940 sponge. It has been validated that MAPK1 can mediate the proliferation as well as the metastasis, so as to regulate the progression of tumor^27,28^. The regulatory effect of hsa_circ_101237 on MAPK1 was confirmed via many experiments including RNA pull-down assay, western blot assay, luciferase assay, as well as qRT-PCR assay. In line with the functional test, hsa_circ_101237 over-expression posed an obvious impact on reversing how hsa_circ_101237 kncok down inhibited the proliferation, the migration, and the invasion abilities owned by NSCLC cells. Above data helped to confirm that hsa_circ_101237/miR-490-3p/MAPK1 regulatory network existed in NSCLC. Meanwhile, there are some limitations in our research, as hsa_circ_101237-miR-490-3p axis might also plays key role in enhancing NSCLC progression by targeting other target genes, which is worthy of further exploration in the future.

To sum up, hsa_circ_101237 can facilitate the progression of NSCLC. The study stressed on a mechanism through which hsa_circ_101237 posed a positive effect on the growth and the metastasis of NSCLC cell. It is necessary to target hsa_circ_101237 as a possible method to treat NSCLC.

## Supplementary information

Supplementary information.
