# Supplementary material for: CircRNA_101237 promotes NSCLC progression via the miRNA-490-3p/MAPK1 axis
Source: Sci Rep. 2020 Jun 3;10:9024. doi: 10.1038/s41598-020-65920-2 (PMC7270109; doi:10.1038/s41598-020-65920-2)

**CircRNA_101237 promotes NSCLC progression via the miRNA-490-3p/MAPK1 axis**

Zhi-ye Zhang, Xiao-hui Gao, Ming-ying Ma, Chun-ling Zhao, Ya-li Zhang, Shuang-shuang Guo

Department of Oncology, The First Affiliated Hospital of Henan University of Science and Technology, Henan province, 471000, China

#Correspondence to: Shuang-shuang Guo, E-mail: guoshuangshuang_1981@126.com, The First Affiliated Hospital of Henan University of Science and Technology, the department of oncology, No. 24, jinghua road, jianxi district, luoyang city, henan province, Zip code 471000

**Supplement FigureS1**

Based on the qRT-PCR analysis on miR-490-3p expression, miR-490-3p presented decreased in NSCLC cells. *P < 0.05; **P < 0.01; ***P < 0.001.


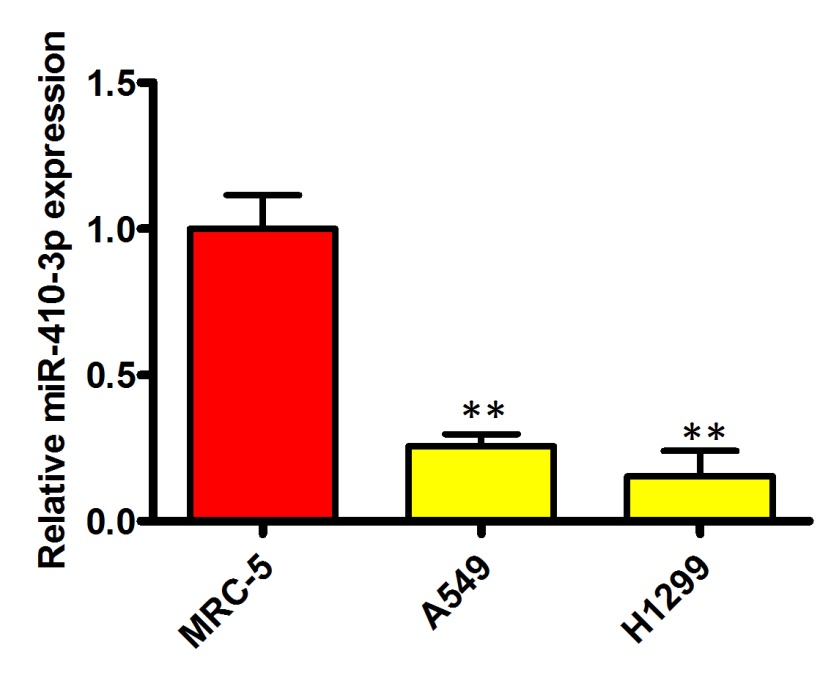


**Supplement FigureS2**

A549 and H1299 cells were co-transfected with si-CircRNA_101237 and miR-490-3p inhibitor used in the experimental research. (A) Cell proliferation was analyzed using the CCK-8 assay. (B, C) Transwell assays of the migratory and invasive abilities of treated NSCLC cells. *P < 0.05; **P < 0.01; ***P < 0.001.

**
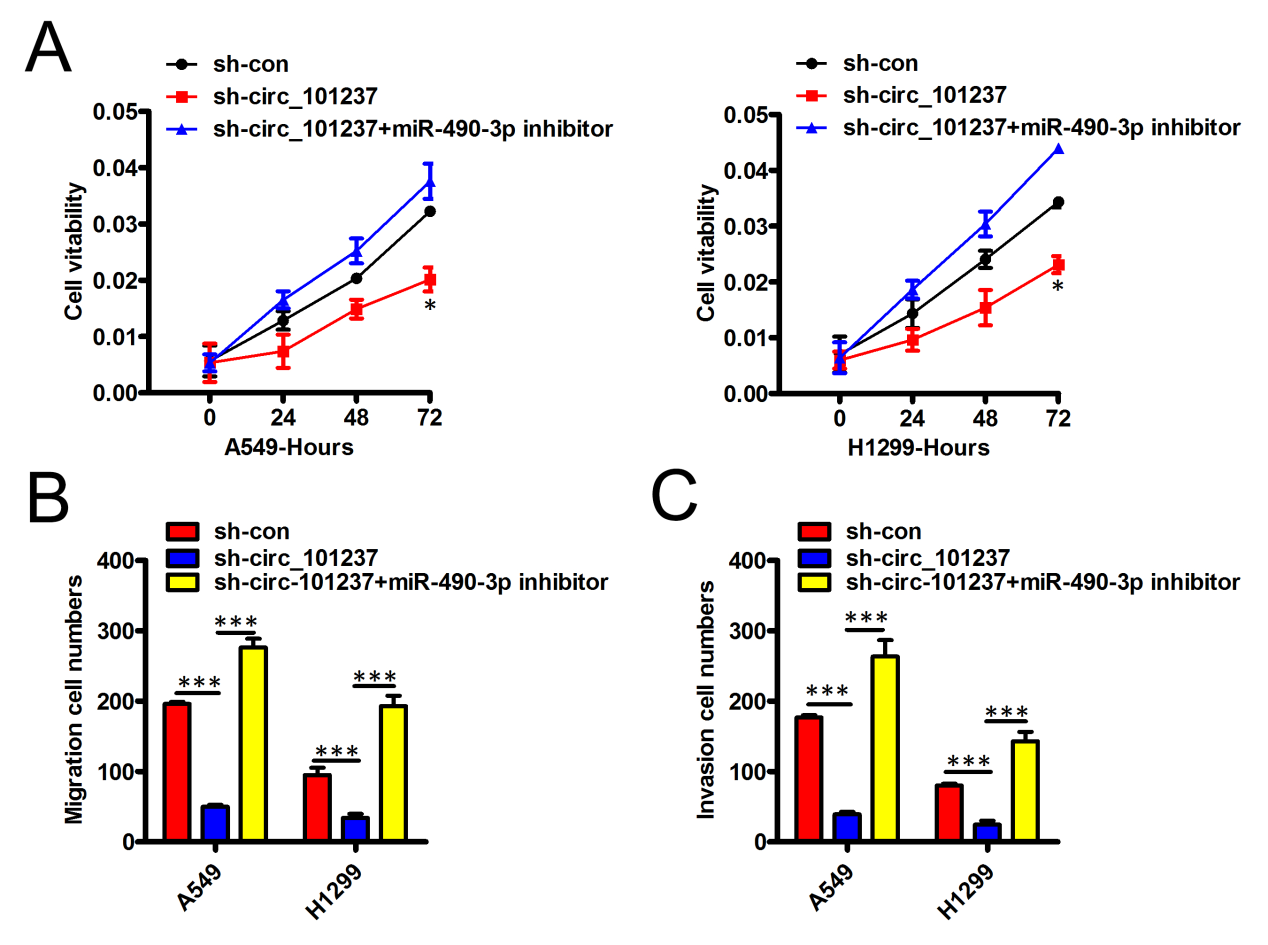
**

**Supplement FigureS3**

(**A** and **B**) The prognosis presented by NSCLC patients whose MAPK1 levels were different was assessed via Kaplan–Meier survival analysis. The cutoff value referred to median MAPK1 expression level in those NSCLC tissues.


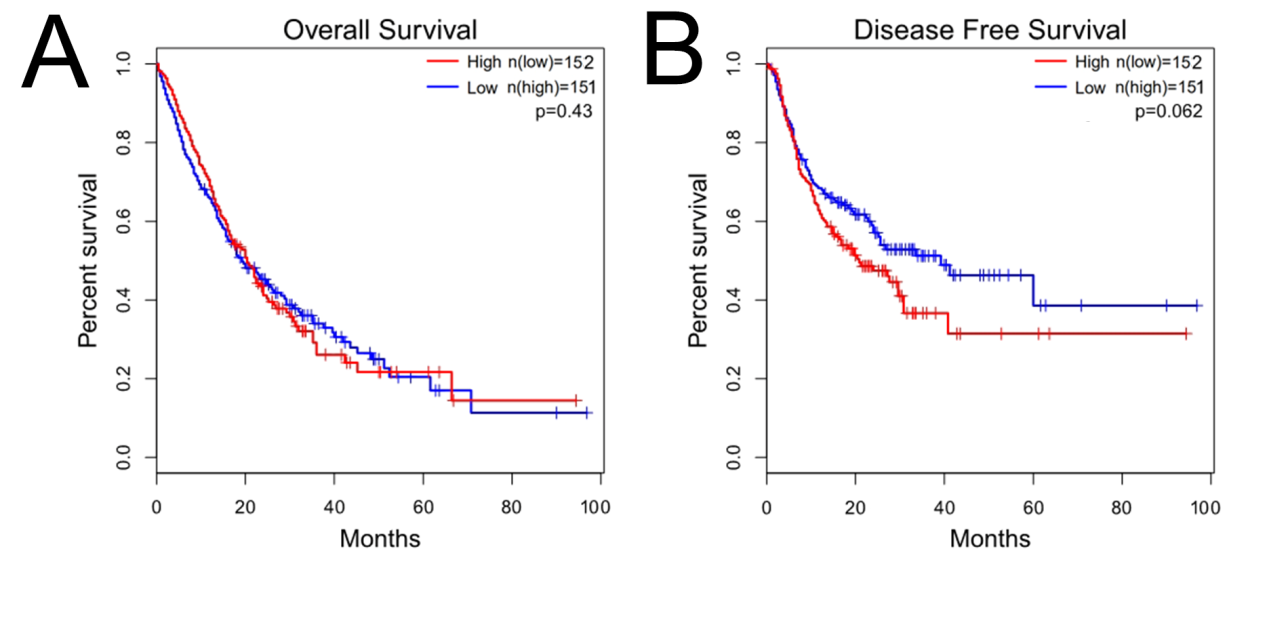

Supplement: Supplementary file 1 — Supplementary information. [file 41598_2020_65920_MOESM1_ESM.docx]
